# The Blocking of Drug Resistance Channels by Selected Hydrophobic Statins in Chemoresistance Human Melanoma

**DOI:** 10.3390/biom13121682

**Published:** 2023-11-21

**Authors:** Wojciech Placha, Piotr Suder, Agnieszka Panek, Patrycja Bronowicka-Adamska, Marta Zarzycka, Małgorzata Szczygieł, Jacek Zagajewski, Monika Weronika Piwowar

**Affiliations:** 1 Department of Biochemistry, Faculty of Medicine, Jagiellonian University Medical College, Kopernika 7b St., 31-034 Krakow, Poland; patrycja.bronowicka-adamska@uj.edu.pl (P.B.-A.); marta.zarzycka@uj.edu.pl (M.Z.); j.zagajewski@uj.edu.pl (J.Z.); 2 Department of Analytical Chemistry and Biochemistry, Faculty of Materials Science and Ceramics, AGH University of Science and Technology, 31-007 Krakow, Poland; piotr.suder@agh.edu.pl; 3 Institute of Nuclear Physics Polish Academy of Sciences, 31-342 Krakow, Poland; agnieszka.panek@ifj.edu.pl; 4 Department of Biophysics and Cancer Biology, Faculty of Biochemistry Biophysics and Biotechnology, Jagiellonian University, 31-007 Krakow, Poland; gosia.szczygiel@uj.edu.pl; 5 Department of Bioinformatics and Telemedicine, Faculty of Medicine, Jagiellonian University Medical College, Kopernika 7e St., 31-034 Krakow, Poland; monika.piwowar@uj.edu.pl

**Keywords:** glycoprotein P, hydrophobic statins, docetaxel, multidrug resistance, melanoma

## Abstract

Despite the development of modern drugs, drug resistance in oncology remains the main factor limiting the curability of patients. This paper shows the use of a group of hydrophobic statins to inhibit drug resistance (Pgp protein). In a chemoresistance melanoma cell model, viability, necroptosis with DNA damage, the absorption of the applied pharmaceuticals, and the functional activity of the ABCB1 drug transporter after administration of docetaxel or docetaxel with a selected hydrophobic statin were studied. Taxol-resistant human melanoma cells from three stages of development were used as a model: both A375P and WM239A metastatic lines and radial growth phase WM35 cells. An animal model (*Mus musculus* SCID) was developed for the A375P cell line. The results show that hydrophobic statins administered with docetaxel increase the accumulation of the drug in the tumor cell a.o. by blocking the ABCB1 channel. They reduce taxol-induced drug resistance. The tumor size reduction was observed after the drug combination was administrated. It was shown that the structural similarity of statins is of secondary importance, e.g., pravastatin and simvastatin. Using cytostatics in the presence of hydrophobic statins increases their effectiveness while reducing their overall toxicity.

## 1. Introduction

The phenomenon of multidrug resistance (MDR) is one of the main causes of systemic cancer therapy failure. Some tumors show primary drug resistance. However, in the vast majority of neoplasms, neoplastic cells acquire the characteristics of drug resistance during chemotherapy. Initially, a neoplasm that is sensitive to the drug administered may acquire a drug-resistant phenotype due to incorrect dosages or changes in the bioavailability or metabolism of the drug. Multi-drug resistance is defined as the acquisition by tumor cells of simultaneous insensitivity to several groups of different, unrelated therapeutic agents that develop in response to the administration of a single cytostatic drug. The phenomenon of drug resistance, as mentioned, may result from many different pharmacological and physiological conditions. Pharmacological factors triggering drug resistance include incorrect dosage, changes in the bioavailability of the drug, or modification of cell metabolism. At the cellular level, the formation of multidrug cross-resistance in neoplastic cells is associated with a change in the rate of drug entry into the cell and drug transport between the nucleus and the cytoplasm. An important mechanism may also be changed in the amount and affinity of enzymes responsible for drug metabolism, e.g., the participation of cytochrome P450 in the activation or inactivation of pharmacological compounds [1]. One should not forget about the differences in the metabolism of neoplastic cells compared to the original cells for a given type of neoplasm. They manifest as the ability to disturb the processes of apoptosis regulation, damage DNA repair, or use “atypical” metabolites. Also, particularly important in anticancer therapy is the possibility of the active removal of cytostatics from cells by transporter proteins. The latter role is mainly attributed to transmembrane transporters from the ABC protein group (ATP-binding cassette transporters). The ABC family of protein transporters is common in the animal kingdom, including *Homo sapiens* [2]. It consists of membrane proteins involved in the transport of many substances (mainly hydrophobic) through intra- and extracellular membranes, using energy from ATP hydrolysis. Their most important function is participation in detoxification and protection of the body against poisons. The consequence of these protective functions, as previously mentioned, is the development of the multi-drug resistance phenomenon, through the active removal of cytostatics (and other drugs) from tumor cells, which makes it impossible to achieve the appropriate concentration of drugs in the treated tissues. Currently, intensive research is being carried out on the development of new therapies that take into account the MDR phenomenon [3]. The ABC superfamily includes various important proteins associated with cancer resistance to cytostatics, such as multidrug resistance protein 1 (MRP1 and ABCC1), breast cancer resistance protein (BCRP and ABCG2), glycoprotein P (Pgp and ABCB1) [4], and ABCB5. The latter is one of the most important proteins involved in resistance to cytostatics. High expression of Pgp has been found on the surface of cells that perform secretory functions: in the adrenal cortex, in the proximal epithelium of the renal tubules and pancreatic ducts, or in the liver bile duct cells. The ABCB1 protein, contributing to the formation of MDR, is present in many types of cancer, especially high expression in tumors characterized by resistance to chemotherapeutic agents [5]. A classic example of such a tumor is melanoma [6]. Relatively high Pgp expression occurs in the stem cells of the hematopoietic system [7]. Its expression has been shown to decrease during stem cell development and differentiation into mature target blood cells. Interestingly, also in neoplastic tumors, e.g., human melanoma, a significant amount was found, up to 30% of the mass of the so-called malignant stem cell-like (CSCL) cells. The mechanisms of tumor recurrence caused by CSCL cells have been well described in leukaemia and solid tumors [8]. In addition to melanoma, they have been described in colorectal adenocarcinomas, pancreatic and glioblastoma cancers, and primary liver cancer. The phenomenon of overexpression of ABC proteins in CSCL cells and “somatic tumor” cells which translates into cancer therapy failure is widely described in the literature [9]. Pgp is encoded by the MDR1 gene located on the long arm of chromosome 7 (7q21). This protein consists of 1280 amino acids and its mass is equal to 170 kDa. The secondary structure of Pgp consists of two homologous subunits containing transmembrane domains (TMDs) and polar nucleotide-binding domains (NBDs). The nucleotide-binding domain contains two sites responsible for ATP hydrolysis (Walker A and Walker B motifs). At least two sites in the hydrophobic sequence region are responsible for drug binding (DBS) [5]. The Pgp substrate spectrum includes various xenobiotics, including cytostatics. These are mainly hydrophobic compounds, including Vinca alkaloids (vincristine, vinblastine, and their semi-synthetic derivatives), taxanes (paclitaxel and docetaxel), colchicine, and mitomycin C, as well as puromycin, etoposide, and anthracyclines (doxorubicin, daunorubicin, and actinomycin D) [10]. Contemporary oncology has at its disposal a large number of chemical compounds used in anti-cancer therapies. One of these numerous groups of chemotherapeutic agents is compounds whose mechanism of action is directed against microtubules. Microtubules are cellular protein polymers that build the cytoskeleton and are present in all eukaryotic cells. They are composed of α- and β-tubulin heterodimers (MW dimer approx. 100 kDa), which combine in tandem to form protofilaments [11]. The result of this type of zpolymerization is the presence of filaments of two poles in the “tubes”, differing in the kinetics of attachment and removal of heterodimers from individual ends. The polymerization process requires energy from GTP hydrolysis and is reversible. As a result of the association of usually 13 protofilaments, a microtubule is formed in the shape of a “hollow cylinder”, the length of which is several micrometers. These compounds disrupt the operation of the karyokinetic spindle, thus preventing cell division. The course of mitosis disrupted by the drug leads to cell cycle arrest (on the border of metaphase and anaphase), directing the cell to the path of physiological death manifested as a mitotic catastrophe [12], necroptosis, or necrosis. The legitimacy of using “mitotic poisons” in oncological therapy is confirmed by the fact that the frequency of division of most neoplastic cells in the entire tumour mass is higher than in normal cells. This translates into increased sensitivity to these compounds. As a rule, however, the so-called “Somatic tumour cells” are killed. CSCL cells effectively clear drugs from the cytoplasm while remaining in the G0 phase [8]. Drugs from this group can bind to the dissolved form of tubulin and/or to tubulin directly forming part of the cytoskeleton. When used in high concentrations of 10–500 nM, they lead to the destruction of the division spindle structure, thus stopping mitosis in dividing neoplastic cells. Two general classifications of drugs act on the spindle. The first was created due to the binding site of a given compound to a specific region of the tubulin heterodimer. Taking this division into account, Vinca alkaloid, paclitaxel, and colchicine binding sites in specific domains of the protein are distinguished. The second group of antimicrotubule substances contains compounds that change the dynamics of microtubules. However, drugs from this group are also associated with the occurrence of multi-drug resistance (MDR). Currently, the attention of many research teams is focused on breaking the above-mentioned therapeutic limitations. New semi-synthetic taxanes such as DJ-927, XRP6258, and XRP9881 have been developed [13]. They show greater antitumor activity, lower toxicity, increased penetration of the blood–brain barrier, and better solubility in water. Despite the MDR phenomenon, we can talk about the clinical success of drugs from the taxane group. This has led to the discovery of other compounds that enhance microtubule polymerization. These include epothilones, diskodermolide, eleuterobin, and laulimalide, which are less sensitive to MDR [14,15,16,17]. However, there are different therapeutic approaches. The channels responsible for MDR in cancer cells can be inhibited. Various compounds that can influence the function of ABC proteins are currently being sought. The activity of protein transporters, responsible for the ejection of cytostatic molecules from the inside of the cell, may be stopped at several levels of its functioning. At the transcription level, expression can be inhibited by modifying the gene’s promoter or blocking the factors involved in its transcription. In the mechanism of interaction with the channel protein, by inhibiting the activity of transporters, it abolishes the MDR of cancer cells. Such molecules are called chemo sensitizers, MDR inhibitors, or modulators [18]. They belong to many groups of chemical compounds and show similar structures—high hydrophobicity, low molecular weight, and the presence of an aromatic ring in the molecule [19]. There are three generations of Pgp inhibitors based on the mechanism of action and affinity for the target transporter protein. First-generation inhibitors, cyclosporin A, erythromycin, or verapamil, are drugs that, in the structure of the molecule, are very similar to the substrates of target transporters [20,21,22]. However, due to the low affinity to ABC proteins, the effectiveness of this generation of drugs requires their administration in high doses, which leads to acute toxicity [23]. Second-generation inhibitors such as R-verapamil, dexniguldipine, and valspodar are characterized by lower toxicity and a better pharmacological profile, but they still have an insufficient affinity to Pgp. Due to the shortcomings of the previous generations of drugs in question, the third generation of inhibitors was developed. They have a high affinity to Pgp and have a low risk of binding to other transporters and disrupting their function. They can be administered in low concentrations but are pharmacologically active. They bind to the target protein in an incompetent manner, forcing a conformational change in the transporter molecule and inhibiting its activity. This leads to the release of cytostatic molecules outside the cancer cells. The group of third-generation inhibitors includes substances such as tariquidar, biricodar, annamycin, mitotane, and laniquidar [24]. There are also a number of molecules that more or less specifically inhibit Pgp. Among them are also statins [25,26,27]. However, there are reports of an ambiguous role of statins in this process. Some molecules/drugs are able to brake others are not. Cancer therapies using conventional cytostatic drugs do not work on “cancer stem cells” as mentioned. As a rule, cytostatics destroy the dedifferentiated or differentiating tumour cells. Somatic tumour cells are unable to form new disease outbreaks. On the other hand, CSCL cells give rise to new tumours as they divide. They are the ones that survive the cytostatic therapy, which is the reason for the recurrence of the neoplastic disease. Therefore, the aim of anti-cancer therapy targeting “stem cells” is those therapy variants that block drug resistance, which CSCL cells in particular exhibit. This leads to the “awakening” of the tumour CSCL cells from the G0 phase and the transition to the cell division phase to avoid unfavourable factors in the environment. Such a mechanism of action was proposed by the authors of the study by administering docetaxel to melanoma cells (as a model of a tumour that is highly resistant to chemotherapeutic agents), with a specific modulator of the Pgp channel—hydrophobic statins. By blocking or weakening the activity of Pgp, the efficiency of the therapy can be increased. It allows for the return of previously used pharmaceuticals to which cells quickly developed resistance. This gives a chance for a non-specific impact on CSCL cells in neoplastic tumours as well.

## 2. Materials and Methods

### 2.1. Chemicals and Reagents

Simvastatin, pravastatin, rosuvastatin, fluvastatin, and atorvastatin were purchased from (Merck, Darmstadt, Germany). Docetaxel (Calbiochem Merck, Darmstadt, Germany), and physiological salt—The Infusion Fluids Manufacturing in Kutnio, Poland. Trifluoroacetic acid (TFA), 2-propanol, chloroform, acetone, and DMSO were obtained from Sigma (Schelldorf, Germany). Gradient-grade acetonitrile (ACN) was purchased from Merck (Darmstadt, Germany). Water was deionized by passing it through an Easy pure RF compact ultrapure water system.

### 2.2. Cell Cultures and Preparation of Biological Material

Experiments were carried out using cell lines of human melanoma originating from three stages of development: WM35 from the radial growth phase, a WM239A metastatic line, and A375P metastasis from the lungs, a malignant line. The cell lines were obtained from Trust Functional Genomics Cell Bank, London, UK. The cells were grown in a monolayer in RPMI culture medium (ThermoFisher Scientific, Waltham, MA, USA) supplemented with 10% fetal bovine serum (EURx, Gdansk, Poland) in Petri dishes (100/60/mm diameter, Corning, Arizona, San Diego, CA, USA) at 37 °C, in a humid atmosphere with 5% CO_2_ content in normoxic (21% O_2_) conditions. Cultivation was carried out without antibiotics. All of the melanoma cells tested negative for mycoplasma when using a MycoCheckTM Mycoplasma PCR Detection Kit (MoBiTec Molecular Biotechnology, Goettingen, Germany). For the MTT (and reference crystal violet) viability, GSH content, and HPLC drug uptake experiments, all three cell lines were cultured for 10 passages in sub-lethal doses of 1 nM docetaxel. After this time, cells of individual lines were detached from the substrate, counted, and plated on 96-well plates at 1.5 × 10^3^ per well. The cells were counted using a Countess Invitrogen TM counter. The cells were cultured for 48 h in the presence of drugs in appropriate compositions and concentrations—the same for all lines, i.e., A375P, WM239A, and WM35: control—10% fetal bovine serum (FBS) in RPMI medium (Roswell Park Memorial Institute), 0.5 μM docetaxel in medium, statins (simvastatin, rosuvastatin, fluvastatin, pravastatin, and atorvastatin), varying from 2.5–100 μM or 20 μM, docetaxel 0.5 μM + the appropriate statin (simvastatin, rosuvastatin, fluvastatin, pravastatin, and atorvastatin), varying from 2.5–100 μM (6 measurement points).

### 2.3. Performing the MTT Test

Absorbance was measured at the wavelength of 570 runs (Synergy HT Bio-Tek spectrometer). Merck’s optimized MTT test kit was used following the manufacturer’s protocol. There were 24 technical replications per measuring point.

### 2.4. Determination of Protein Concentration

The description is in the Supplementary Materials.

### 2.5. Pgp Protein ATPase Activity Test

The ATPase activity assay was performed using the ADP-Glo Max Assay following the manufacturer’s protocol (Promega Corporation, Madison, WI, USA). Other materials used: purified membranes overexpressing the Pgp protein obtained in a heterologous Sf9 insect cell system infected with a recombinant baculovirus transfected with this protein (gift from Csilla Özvegy-Laczka of the Biomembrane Research Group, led by Balazs Sarkadi, Budapest, Hungary), and docetaxel, atorvastatin, fluvastatin, rosuvastatin, and simvastatin (Sigma-Aldrich, St. Louis, MO, USA). Each of these statins was tested at three concentrations: 1 µM, 2.5 µM, and 5 µM. The control was the ATPase activity of the Pgp transporter in the presence of docetaxel but without statins. The specificity of the reaction was assessed by comparing the level of ATP degradation in the system with the Pgp protein and docetaxel versus the system without the Pgp protein. Measurements were performed on Corning™ 96-Well White Polystyrene Microplates (Corning Incorporated, New York, NY, USA) dedicated to luminescence; measurements were made using a Tecan Infinite 200 Pro microplate reader equipped with a luminescence detector (Tecan, Männedorf, Switzerland).

### 2.6. Microscopic Test of the Annexin V-Propidium Iodide Staining of Apoptotic Cells

The description is in the Supplementary Materials.

### 2.7. Evaluation of the Genotoxicity of the Drugs Used in Melanoma Cells

#### 2.7.1. Description of Experimental Groups

The assessment of the genotoxicity of the drugs used in melanoma and colorectal cancer cells was carried out by assessing the level of DNA damage, obtained using the alkaline comet technique. The study assessed the degree of DNA damage in 3 cell lines, melanoma lines A375P, WM239A, and WM35. For each cell line, the level of DNA damage was assessed in cells not treated with the test compounds, cells treated with 0.5 μM of docetaxel only, and cells treated with 0.5 μM of docetaxel and 20 μM of simvastatin, fluvastatin, and atorvastatin. The level of DNA damage was assessed using the Komet 3.0 program and the visual method based on the Gedik classification. The data obtained for the tested cell lines based on the analysis with the Komet 3.0 system are presented below.

#### 2.7.2. Comet System 3.0. Quantitative DNA Damage Analysis [28,29] (Description in the Supplementary Materials)

Determination of the level of reduced glutathione (GSH) and oxidized glutathione (GSSG) was achieved through the use of RP-HPLC (reverse phase high-performance liquid chromatography). The study was carried out on 3 human melanoma cell lines, A375P, WM239A, and WM35. For each cell line, the level of GSH and GSSG was assessed in cells not treated with the test compounds, cells treated with 0.5 μM of docetaxel only, and cells treated with 0.5 μM of docetaxel plus simvastatin, rosuvastatin, atorvastatin, pravastatin, and fluvastatin at a concentration of 20 μM. The preparation of samples for RP-HPLC analysis and the applied analysis using the RP-HPLC method are described in [30].

### 2.8. Analysis of the Absorption of Pharmaceuticals into Human Melanoma Cells

The studies were performed on human melanoma cell lines from three developmental stages: WM35 from the radial growth phase, a WM239A metastatic line, and A375P melanoma metastasis isolated from the lungs, a malignant line. For each measurement point for each of the separate cell lines, cells were cultured on 4 dishes (Corning) of 100 mm in diameter. Analyses were performed for the control system (cells cultured in the presence of 10% FSC serum in RPMI medium), with the addition of 0.5 µM docetaxel and with the addition of the composition: 0.5 µM docetaxel + 20 µM statin (atorvastatin, fluvastatin, rosuvastatin, and simvastatin). The analyses were repeated 5 times in independent experiments with similar results. The results were averaged and reported in µM per million cells. The cells were counted using a Countess Invitrogen TM counter. The preparation of samples for RP-HPLC analysis and the applied analysis using the RP-HPLC method are described in [31]. Mass spectrometry analysis of the selected active compounds was carried out (description in the Supplementary Materials).

### 2.9. In Vivo Studies in SCID Mice

The studies involving human/animal participants were reviewed and approved by resolution No. 259 121/2018 of 28 March 2018, 1st Local Ethical Committee for Animal Experiments in Krakow, Poland. The experiment consisted of two stages. Stage I: selection of the cell line to be inoculated (see details in the Supplementary Materials). Stage II: comparison of the effectiveness of the docetaxel/simvastatin therapeutic composition by determining the effect on the kinetics of tumor growth on the selected A375P human melanoma cancer line. The effectiveness of docetaxel/simvastatin combination therapy compared to docetaxel monotherapy was studied. Three experimental groups were planned: mice (*Mus musculus* SCID) treated with docetaxel (monotherapy model) *n* = 15 in both experimental groups, total *n* = 15 animals; mice treated with docetaxel and simvastatin (combination therapy model I) *n* = 15 in both experimental groups, total *n* = 15 animals; and saline-treated mice (control—no treatment) *n* = 10 in both experimental groups, total *n* = 10 animals. In total, 58 animals (males and females) were planned for both research stages. The size of the above experimental groups was calculated using the following formula: $n=1+2C({\frac{SD}{d})}^{2}$, where: *C* = 10.51 (at α = 0.05 and β = 0.9), *d*—assumed minimum difference, and SD—standard deviation. Each animal was intravenously administered 100 µL of a solution of the therapeutic composition of docetaxel in saline at a concentration of 17 µM/kg body weight (BW) (group of *n* = 15, line A375P) or docetaxel and simvastatin at concentrations of 17 µM/kg BW and 50 mg/kg body weight (group *n* = 15, line A 375P), respectively, and saline for the controls (group *n* = 10, line A375P). Before the procedure, the animal was transferred to a special heating platform at 37 °C for about 10 min to better visualize the tail blood vessels. The mouse was then immobilized in a special injection container for approximately 15–20 s. Administration of the medicated/saline composition was scheduled for the time point at which the tumor reached 4–5 mm in diameter. Three administrations of the above-indicated doses were injected at five-day intervals from the first injection.

### 2.10. Statistical Methods

The analysis and data visualization were carried out in the statistical environment R using the “stats” library [32]. To compare the mean values in the groups, a one-way analysis of variance (ANOVA) was carried out. To show the difference between the groups, the Tukey’s post hoc test was used. 

## 3. Results

### 3.1. Effect of Docetaxel and the Combination of Docetaxel with Statins on Melanoma Cell Viability (MTT Test)

A synergistic effect of docetaxel administered with an appropriate statin on the viability of human melanoma tumor cells was hypothesized. The hypothesis was tested on melanoma cells from three developmental stages: WM35-radial, WM239A, and A375P metastatic lines. Originally, the viability effect of the docetaxel/statin composition was tested using a high-sensitivity assay (crystal violet assay). Varying statin concentrations were the control for the docetaxel/statin formulation, where statins were also given at varying concentrations. 

**Figure 1:**
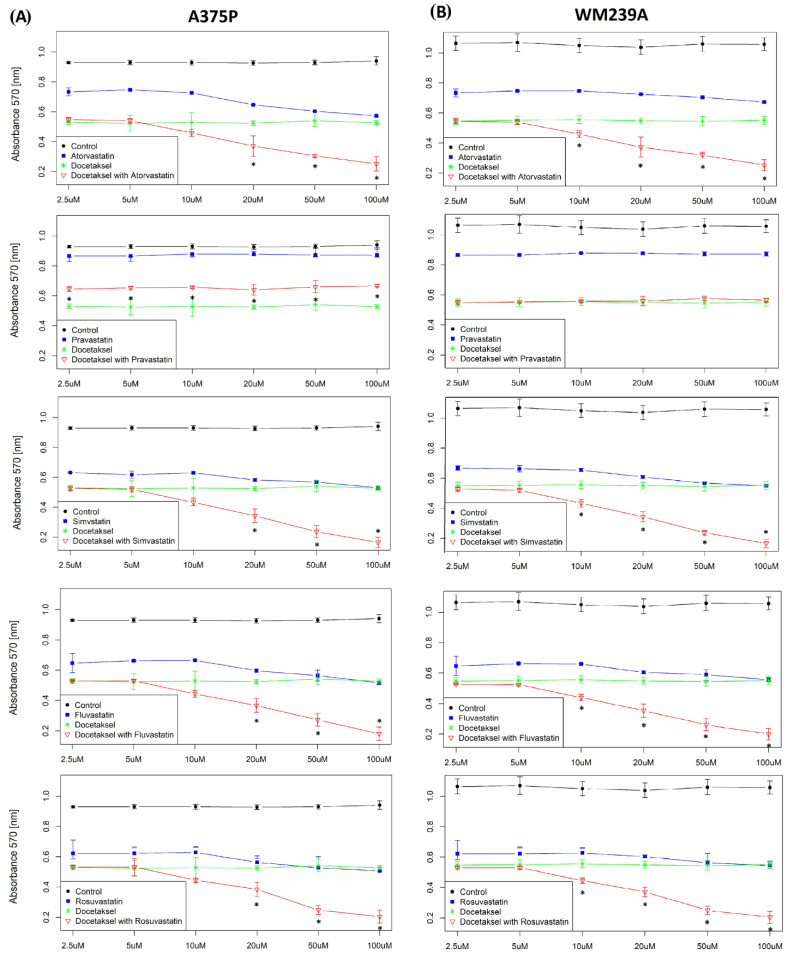
Effect of selected statins administered with docetaxel on human melanoma. (**A**,**B**) MTT test result—cell viability of metastatic melanoma (chemoresistance) lines A375P and WM239A. The cell lines were cultured for 10 passages in sub-lethal doses of 1 nM docetaxel. After this time, cells of individual lines were detached from the substrate, counted, and plated on 96-well plates at 1.5 × 10^3^ per well. The cells were cultured for 48 h in the presence of drugs in appropriate compositions and concentrations. Control—10% FBS in RPMI, docetaxel—0.5 μM in medium (constant concentration), and statins (simvastatin, rosuvastatin, fluvastatin, pravastatin, and atorvastatin)—varying from 2.5–100 μM, docetaxel 0.5 μM (constant concentration) + appropriate statins, varying from 2.5–100 μM (six measurement points). The absorbance was measured in the range of 570 nm. There were 24 technical replications per measuring point. ANOVA tests were used for statistical analysis. Significant results are marked by asterisks.

On this basis, the concentration of statins (20 μM) was selected as the concentration with a sufficient dose acting on melanoma cells and analogously the concentration of docetaxel (0.5 μM). On this basis, an experiment using the MTT test was designed. Docetaxel (green line) was administered as a control at 0.5 µM, and statin at 2.5–100 µM (purple line) (Figure 1 and Figure S1). All of the test samples contained docetaxel at a concentration of 0.5 µM with the corresponding statin in a variable concentration range: from 2.5–100 µM (red line). In all tested cell lines, using both tests, the synergy of the action of the docetaxel composition administered with a hydrophobic statin was demonstrated (Figure 1 and Figure S1). Only pravastatin did not show synergism of action. The degree of inhibition of viability was similar in all three cell lines. Importantly, statins alone showed inhibition of viability, but less so than docetaxel administered alone. The lack of effect of pravastatin on viability was the basis for excluding it from further analyses.

### 3.2. Effect of Docetaxel and the Combination of Docetaxel with Statins on the Necroptosis of Metastatic Melanoma Cells

A hypothesis was made about the effect of the docetaxel–hydrophobic statin composition on inducing the necroptosis process in melanoma cells. To this end, early-stage necroptosis was tested using the annexin/propidium iodide assay and analyzed under a fluorescence microscope. 

**Figure 2:**
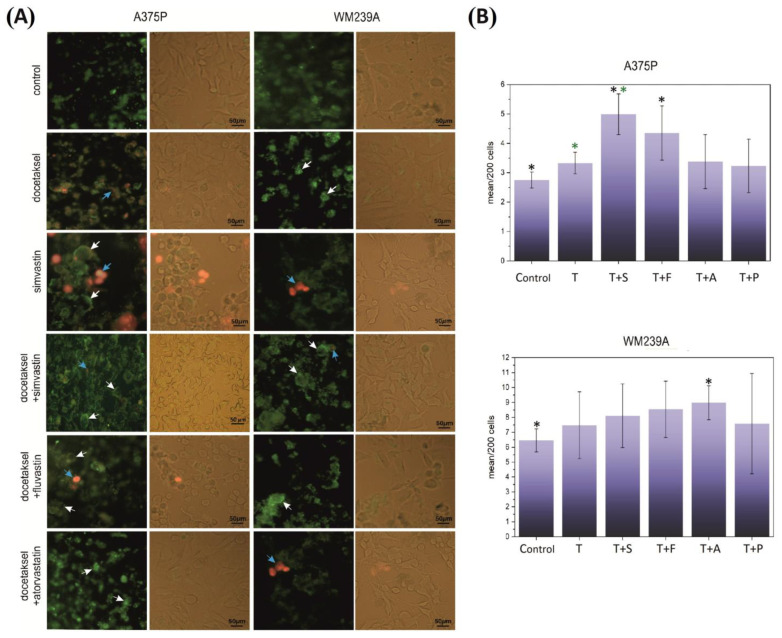
(**A**) Effect of docetaxel and the combination of docetaxel with statins on the necroptosis of metastatic melanoma chemoresistance cells presented on a fluoresce image and a transmitted light image with use of the annexin and propidium iodide staining test. Chemoresistance cells were cultured on a microscope slide in small plates for 24 h, then stimulated with drugs at the above-mentioned concentrations (docetaxel 0.5 μM and statins 20 μM) for 24 h. The excitation wavelength for fluorescein (with annexin) is 488 nm, and the emission maximum is 518 nm. For propidium iodide: 488–540 nm and 617 nm, respectively. Cells not stimulated with the tested drugs were used as a negative control (melanoma cells cultured for 24 h). For example, white arrows indicate necroptosis, and blue arrows indicate necrosis. (**B**) Comparison of DNA damage in the comet assay test for melanoma cell lines (A375P and WM239A) in which statins were used together with taxol. The TM value (tail moment length) was used for the calculations. An average of 200 cells was used in the experiment. The results are the average of 11 independent repetitions. ANOVA and Tukey’s post hoc tests were used for statistical analysis. Significant results were marked by asterisks. Black asterisks indicate significance relative to Control, and green asterisks to T (docetaxel).

The cells of the metastatic lines were treated with simvastatin and fluvastatin as highly hydrophobic and atorvastatin as weakly hydrophobic/a neutral statin. The composition of pharmaceuticals used in all cases (docetaxel and docetaxel with a statin) induced cell necroptosis (Figure 2A). In the A375P line, the microscopic image also showed some involvement of necrosis (fluvastatin and simvastatin with docetaxel and docetaxel administered alone). Necrosis and necroptosis (as evidenced by the movement of phosphatidylserine across the cell membrane) often co-occur with mitotic catastrophe or are a direct consequence of it. The results of the experiments confirm the hypothesis.

### 3.3. Effect of Docetaxel and the Combination of Docetaxel with Simvastatin on the Level of DNA Damage Measured by the Comet Assay

In connection with the results obtained from experiments showing necroptosis in metastatic lines and changes in the GSH/GSSG ratio in cells, a hypothesis was put forward about the increase in DNA damage in the three cell lines studied. The test was performed in the early stages of necroptosis so that the process of already ongoing necroptosis (or necrosis) was not the main source of damage. It was assumed that the analyzed damage would be directly related to the impact of the pharmaceuticals used. There was no significant difference in the level of DNA damage between cells cultured with docetaxel and those cultured with docetaxel and an appropriate statin. Only in the case of the line originating from the WM35 radial phase, the possible protective nature of simvastatin was observed. In all tested lines, the increase in damage after the statin with docetaxel compositions concerning docetaxel was not greater than in the case of docetaxel concerning the control. However, in all cases, an increase in damage relative to the control (medium without pharmaceuticals) was evident (Figure 2B and Figure S2).

### 3.4. Effect of Docetaxel and the Combination of Docetaxel with Simvastatin on the Level of Total Glutathione in the Cells

**Figure 3:**
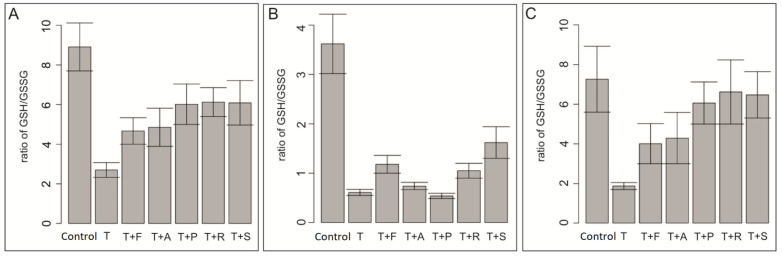
Determination of the level of reduced glutathione and oxidized glutathione in melanoma cell lines. The study was carried out on 3 human melanoma cell lines (**A**) A375P, (**B**) WM35 and (**C**) WM239A,. For each cell line, the level of GSH and GSSG was assessed in: cells not treated with the test compounds, cells treated with docetaxel 0.5 μM only, and cells treated with docetaxel 0.5 μM plus simvastatin, rosuvastatin, atorvastatin, pravastatin, and fluvastatin at a concentration of 20 μM. The preparation of samples for RP-HPLC analysis and the applied analysis took place using the RP-HPLC method. GSH—glutathione reduced; GSSG—glutathione oxidized. A—atorvastatin, F—fluvastatin, P—pravastatin, R—rosuvastatin, S—simvastatin, K—control, and T—docetaxel. The data represent the mean values with the standard deviation. The RP-HPLC technique was used, in 5 replicates. The limit of detection for GSH in the RP-HPLC method is equal to 0.01 [nmol·ml^−1^] and for GSSG 0.1 [nmol·mL^−1^]. The limit of quantification for GSH is equal to 0.1 [nmol·mL^−1^] and for GSSG—1 [nmol·mL^−1^].

To check the cellular toxicity related to the drug composition used and to check the role of glutathione in detoxification processes involving cellular glutathione, its level in cells from three developmental stages of human melanoma was examined. The research hypothesis put forward before the study assumed a significant increase in the level of glutathione, as a result of the conjugated pumping out of the cytostatic from the inside of the cell in all tested cells. As research has shown, a significant increase in the level of reduced glutathione occurred only in metastatic lines. In the WM 35 radial phase line, there was a greater increase in glutathione (GSH) levels compared to docetaxel in the samples stimulated with docetaxel and simvastatin fluvastatin, and rosuvastatin (Figure 3B). Interestingly, an increase in the level of reduced glutathione also occurred in cells cultured in the presence of pravastatin in metastatic cell lines to a similar extent as in the case of co-culture with hydrophobic statins (Figure 3A,C). However, in the WM 35 line, the level of GSH after pravastatin stimulation was the lowest relative to docetaxel alone (Figure 3B). It is worth noting that compared to cells cultured without pharmaceuticals, GSH levels decreased significantly after adding docetaxel to the culture. This decrease applies to cell lines from all developmental stages. Such a decrease in GSH levels can be explained by its increased pumping out of docetaxel cells from the cells, probably with the participation of the glutathione conjugate. The research hypothesis was confirmed only for cells from metastatic phases.

### 3.5. Effect of Docetaxel and the Combination of Docetaxel with Simvastatin on Tumor Growth in Mice Induced from the A375P Cell Line

Under the influence of the results obtained so far regarding metastatic lines, i.e., those that are usually associated with drug resistance, a hypothesis was put forward about the possible impact of the selected composition (simvastatin + docetaxel) on the growth of tumors in an in vivo model. Tumor growth slowdown or remission was assumed with great caution. 

**Figure 4:**
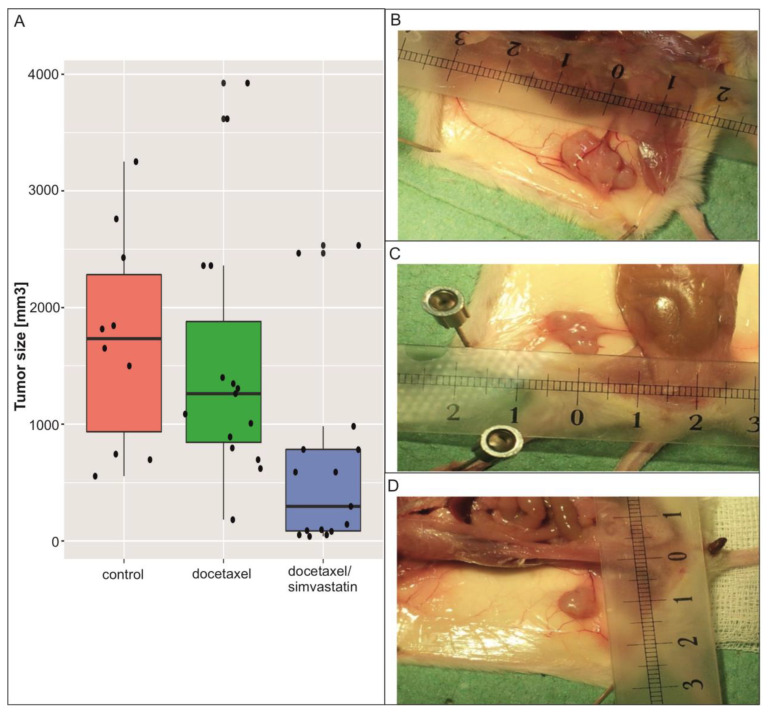
Comparison of the effectiveness of the docetaxel/simvastatin drug composition by determining the effect on the kinetics of tumor growth on the A375P human melanoma cancer line. (**A**) The effectiveness of the composition of two pharmaceuticals, i.e., docetaxel and simvastatin, was tested compared to docetaxel alone. Experimental groups: mice (*Mus musculus* SCID) treated with docetaxel (monotherapy model) *n* = 15 in both experimental groups, in total *n* = 15 animals; mice treated with docetaxel and simvastatin (combination therapy model I) *n* = 15 in both experimental groups, total *n* = 15 animals; and saline-treated mice (control—no treatment) *n* = 10 in both experimental groups, total *n* = 10 animals. In total, 58 animals (males and females) were used for both research stages. The size of the above experimental groups was calculated using the following formula: $n=1+2C({\frac{SD}{d})}^{2}$, where: *C* = 10.51 (at α = 0.05 and β = 0.9), *d*—assumed minimum difference, and SD—standard deviation. Each animal was intravenously administered 100 µL of a solution of the therapeutic composition of docetaxel in saline at a concentration of 17 µM/kg body weight (BW) (group of *n* = 15, line A375P) or docetaxel/simvastatin at concentrations of 17 µM/kg BW and 50 mg/kg body weight, respectively (group *n* = 15, line A375P), and saline for the controls (group *n* = 10, line A375P). ANOVA and Tukey’s post hoc tests were used for statistical analysis. Example photos of melanoma tumors inoculated subcutaneously into SCID mice: (**B**) control mouse, (**C**) docetaxel-treated mouse, and (**D**) docetaxel- and statin-treated mouse.

The SCID mice were inoculated subcutaneously with cells of the potentially most ‘malicious’ metastatic line A375P. The therapeutic composition was administered; post mortem, the size of the tumors was examined. The tumors were slightly reduced in size after the administration of docetaxel. After the use of docetaxel with the simvastatin composition, the weight of the tumors was significantly reduced, and in four cases, complete remission was achieved (Figure 4). Taking into account the median (black cross line in the graph), it can be stated beyond any doubt that the simvastatin with docetaxel composition inhibits the development of tumors in the in vivo model to a much greater extent than docetaxel alone (Tables S1 and S2).

### 3.6. Effect of Docetaxel and the Combination of Docetaxel with Simvastatin on the Action Potential of the ABCB1 Drug Channel

**Figure 5:**
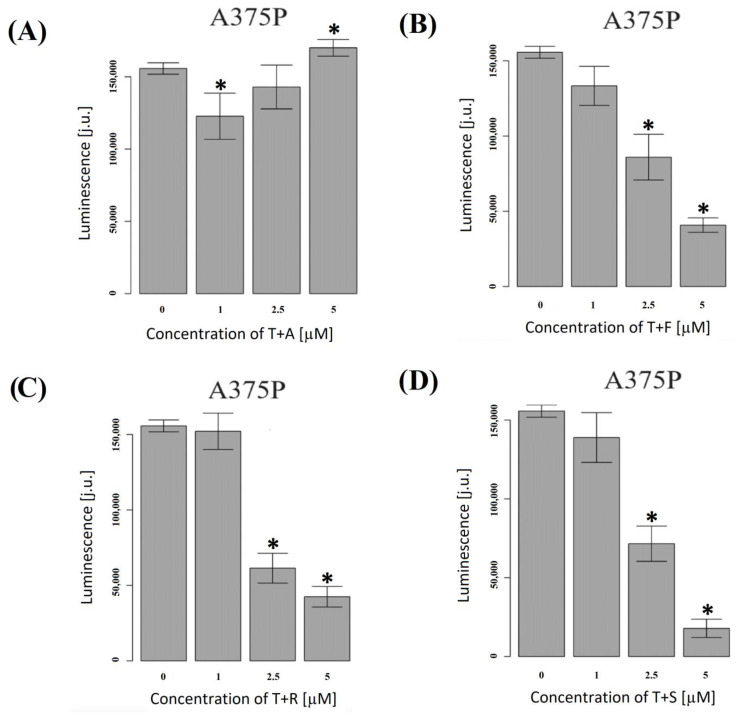
Effect of docetaxel (T) administered with the appropriate statin in the A375P cell line on Pgp drug transporter activity, measured as ATP consumption. (**A**) A—atorvastatin, (**B**) F—fluvastatin, (**C**) R—rosuvastatin, and (**D**) S—simvastatin. The results are the average of 5 independent replications per 8 points. ANOVA and Tukey’s post hoc tests were used for statistical analysis. Significant results are marked by asterisks.

The addition of statins to docetaxel causes a decrease in ATP consumption by the membrane Pgp transporter (Figure 5). In the case of fluvastatin, rosuvastatin, and simvastatin, it was shown that this effect is very clearly intensified with the gradual increase in statin concentration (1, 2.5, and 5 µM). 

The greatest decrease in ATP consumption by the Pgp membrane transporter was observed after using the combination of docetaxel and 5 µM of simvastatin. The results indicate that hydrophobic statins, except atorvastatin, can be modified towards inhibition of the ATPase activity of the Pgp transporter in the presence of docetaxel. Docetaxel is not ejected through the drug channel, which results in a small amount of ATP being used. Atorvastatin is a drug that is more neutral than hydrophobic, which translates into its inability to inhibit the above-mentioned channel (Figure 5A). However, the inhibition of viability and the induction of necroptosis as well as the accumulation of docetaxel in cells indicates that atorvastatin can block other channels than just Pgp drug channels. This indicates a beneficial effect of these statins, especially simvastatin, on P-glycoprotein-mediated docetaxel clearance from cells and, consequently, their impact on the effectiveness of docetaxel chemotherapy.

### 3.7. Recovery of Pharmaceuticals from Human Melanoma Cells

Another hypothesis was put forward about the possibility of accumulating larger amounts of docetaxel inside the cells. To verify it, determinations of docetaxel content in cells of three lines originating from different stages of development of human melanoma were performed. 

**Figure 6:**
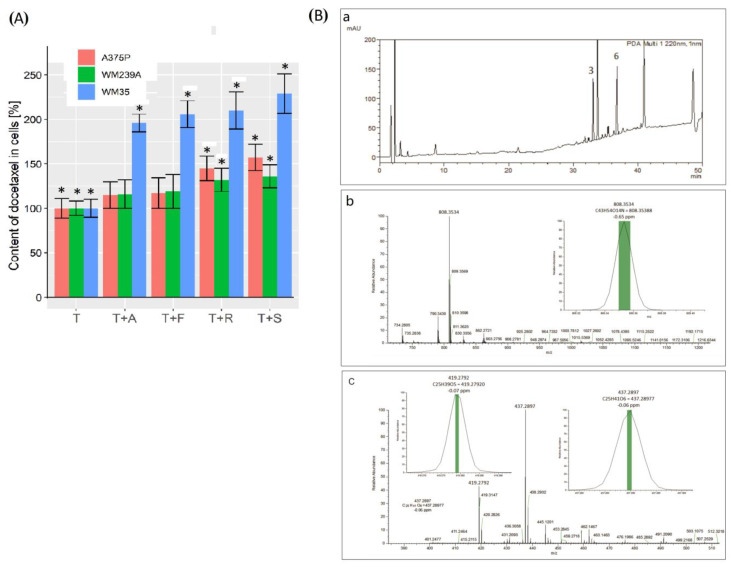
(**A**) The content of docetaxel (T) in human melanoma cells from three developmental stages for lines WM239A, A375P, and WM35. Docetaxel was administered to the cells with the appropriate statin: A—atorvastatin, F—fluvastatin, R—rosuvastatin, and S—simvastatin. The results are the average of 5 independent repetitions of 3 trials each. ANOVA and Tukey’s post hoc tests were used for statistical analysis. Significant results are marked by asterisks. (**B**) RP-HPLC result. (**a**) Example of the analysis of docetaxel uptake into A375P cells modulated with simvastatin (5 independent repetitions of 3 trials each). Peak no. 3 docetaxel; no. 6—simvastatin. (**b**) Mass spectrum of docetaxel. MW = 808.3534 corresponds to (C_43_H_54_O_14_N)1+ ion of the docetaxel molecule. There is a visible part in the insertion close-up view of the monoisotopic peak (error −0.65 ppm). The green bar thickness corresponds to a 1 ppm error. (**c**) Mass spectrum of simvastatin and simvastatin acid ions. Both molecules are visible in the sample, with them staying in equilibrium. Left insertion: simvastatin monoisotopic peak (error −0.07 ppm), right insertion: simvastatin acid monoisotopic peak (error −0.06 ppm). The green bars visible in both insertions represent a 0.2 ppm error.

A composition consisting of docetaxel and successively three different hydrophobic statins were used: atorvastatin, fluvastatin, rosuvastatin, and simvastatin. Contrary to expectations, in cells derived from the radial phase of WM35, docetaxel in the presence of hydrophobic statins were shown to accumulate to the greatest extent (Figure 6A). The metastatic cell lines WM239A and A375P showed less accumulation of docetaxel compared to the radial line WM 35. This result was as expected, but higher content was expected in the metastatic lines. More so the full effectiveness of the docetaxel with simvastatin composition was demonstrated in the in vivo model and the experiments of the ABCB1 channel activity. Despite this fact, the validity of the assumed hypothesis was confirmed. All data obtained from the RP-HPLC absorption analyses were confirmed by mass spectrometry (Figure 6B) (Table S3).

## 4. Discussion

Future research directions may also be drug resistance, both in terms of infectious and oncological diseases, as well as in the case of prolonged therapy with a given drug, which remains the main factor limiting the curability of patients. The problem of drug resistance in neoplastic diseases, associated with uncontrolled cell division, has become the main problem of curability in modern oncology, despite the initial successes of early chemotherapeutics [22]. The original solution to the problem of resistance to single-agent chemotherapy, combined administration of drugs with non-overlapping mechanisms of action or polychemotherapy, was effective in some forms of cancer (breast and testis) and lymphomas [33,34,35,36]. Combination chemotherapy has therefore become the new standard of anti-cancer therapy, which has resulted in the development of more effective multi-drug regimens. Today, however, it can be said that half a century after its introduction, the successes achieved through the use of polychemotherapy have largely stabilized. Surgery, radiotherapy, and polychemotherapy are not enough to cure many types of cancer. The search for new drugs, molecularly targeted at a given cellular mechanism, has begun. This has resulted in the creation of a whole range of modern drugs that significantly improve the results of cancer treatment (e.g., estrogen receptor (ER) and androgen receptor (AR) antagonists, BCR-ABL, HER2, EGFR inhibitors, etc.) [36,37,38]. However, these drugs are not a universal anti-cancer therapy, and many types of cancer develop resistance to them as well [39,40]. It should be noted that, recently, cancer therapy has made a quantum leap using immunological methods, such as anti-CTLA4 12 and anti-PD-1/PD-L1 monoclonal antibodies that inactivate negative regulators or checkpoints of the adaptive immune system [9]. Despite this, drugs that could inhibit multidrug resistance have been sought for a long time. One way is to create new compounds to which cancer cells have not yet acquired resistance. The second method is to directly inhibit the mechanisms responsible for removing drugs from cells. The authors of the study chose the second method, searching among the approved drugs for compounds that could inhibit multidrug resistance. The choice of this method was dictated by the fact that registered drugs often make it possible to shorten the implementation path of a given drug or a given medicinal composition, thanks to the known pharmacokinetic, pharmacodynamic, and toxicological properties of an already registered drug. Among several analyzed groups of drugs (glitazones, sartans, fibrates, and cannabinoids), it was decided based on screening tests to use drugs from the group of statins as potential compounds that could inhibit drug resistance. As mentioned, statins are inhibitors of the enzyme 3-hydroxy-3-methyl-glutaryl-coenzyme A (HMG-CoA) reductase [41]. They also have additional effects, including on the circulatory system, by affecting endothelial function [41,42], stabilizing atherosclerotic plaques [43,44], inhibiting the coagulation system [45,46], stimulating the fibrinolysis system [47], or inhibiting inflammatory reactions, and immunomodulatory effects [48,49]. Statins reduce the number of coronary events, strokes, revascularization procedures, and deaths caused by coronary artery disease [49,50]. It would seem that thanks to these facts, their use in anti-cancer therapy will bring additional benefits. They are relatively well tolerated and their most serious side effect is rhabdomyolysis [51,52]. There are many observations regarding the anticancer effects of statins alone [53,54]. Human melanoma cells from three developmental stages were used as a research model. Cell lines that represent different stages of tumor development in vivo were selected. An animal model was developed for the most aggressive cell line A375P. The cells were implanted subcutaneously into SCID mice lacking an immune system. The tumor size and weight of the animals were assessed. In many types of cancer, tumor size at diagnosis is perhaps the most frequently used variable to assess prognosis. It is assumed that larger tumors correlate with an increased risk of metastasis [55,56]. Melanoma was chosen because of the high prevalence of drug resistance in this tumor. According to the Goldie–Coldman hypothesis, the likelihood that a tumor contains drug-resistant cells depends on the tumor size and mutation frequency [56,57]. However, at a given mutation rate, tumor size becomes a key determinant in predicting the presence of drug-resistant mutations. This model resulted in the concept that it was better to alternate chemotherapy combinations without cross-resistance than to administer all treatments at once. This is often limited by toxicity. This approach becomes better at preventing drug resistance compared to sequential therapies. Although tumour size is a critical determinant of resistance, tumour growth rate and changes in tumour growth kinetics (often induced by therapy) also play important roles in response to therapy and the induction of drug resistance. The Norton–Simon hypothesis is a model that fairly well reflects changes in tumour size after pharmacotherapy [58]. This model, adopted for solid tumours, is based on Gompertz growth curves. According to it, cancerous tumours grow exponentially, faster at a low tumour burden, and then approach a plateau with a slower growth rate as they grow larger (sigmoidal growth curve) [59]. The drugs used reduce the size of tumours and affect the kinetics of their growth. After a single administration of chemotherapy, the remaining fractions of the tumour can regress to an early phase of exponential growth. According to this approach, the likelihood of eradication is maximized by preventing rapid tumour regrowth. However, at a given mutation rate, tumour size becomes a key determinant in predicting the presence of drug-resistant mutations. Docetaxel has been used as a model drug for the treatment of melanoma. This classic chemotherapeutic drug induces cell death through mitotic catastrophe, which is easily observed microscopically and which manifests itself quite rapidly in necroptosis. It is also relatively easy to capture the DNA damage that accompanies the late mitotic catastrophe. These changes were observed with the comet test [60,61]. After statins were selected as potential drug resistance modulators, those that most effectively inhibited cell division were selected using a docetaxel regimen with an appropriate statin. It has been shown that the best effects in the form of inhibition of viability are brought by the use of statins, and only hydrophobic ones. Proliferation/viability (as well as toxicity) was tested using the MTT assay. It is a widely recognized test used in preclinical studies, recommended by drug registration centres. Its great advantage is low sensitivity to changes. This gives a chance to capture specific effects. Nevertheless, screening was performed with the crystal violet assay, an inexpensive, highly sensitive assay (data available from the author and in patents). Melanoma cells were treated with docetaxel and docetaxel administered with a suitable statin. Previously, the cells were grown in sub-lethal concentrations of docetaxel to induce drug resistance. Compared to the hydrophobic statin simvastatin, atorvastatin and fluvastatin, which dose-dependently and synergistically inhibited the proliferation/viability of melanoma cells from three developmental stages, pravastatin had no effect. It was shown that only it was unable to inhibit the viability of melanoma cells. These data are consistent with observations of other authors on other types of cancer. Simvastatin, and not pravastatin, has been shown to inhibit the proliferation of oesophageal adenocarcinoma and squamous cell carcinoma cells [62]. It differs from other statins in its relatively high hydrophilicity [63]. Interestingly, a large randomized trial showed an increase in cancer incidence among elderly people treated with pravastatin [64] (a detailed meta-analysis and meta-regression analysis of randomized controlled trials was performed). Interestingly, functional tests of the multidrug transport channel ABCB1 did not show the ability of pravastatin (data available from the author) and atorvastatin to inhibit this process. In both cases, there was even a slight increase in Pgp activity. According to the literature [65], the ABCB1/Pgp transporter is preferentially expressed in stem cell-like human melanoma cells [66], as is ABCB5. It can be concluded that the expression of ABC transport proteins in melanoma cells capable of self-renewal and differentiation identifies MDR as the mechanism by which melanoma stem cells promote tumour growth [67]. Although the authors of the study did not characterize the stem cell markers in melanoma, based on the data obtained, a cautious conclusion can be drawn that pravastatin promotes the increase in the activity of the ABCB1 channel and thus, indirectly, tumour growth. Caution in making this hypothesis was dictated by the effect of atorvastatin. It was verified [31] by analyzing the absorbed drug into melanoma cells using HPLC, confirmed by mass analysis, that pravastatin was the only one tested that did not increase the absorption of docetaxel into melanoma cells. In the case of the use of atorvastatin with docetaxel, an increased amount of docetaxel was observed in melanoma cells in all cell types derived from different stages of cancer progression. 

**Figure 7:**
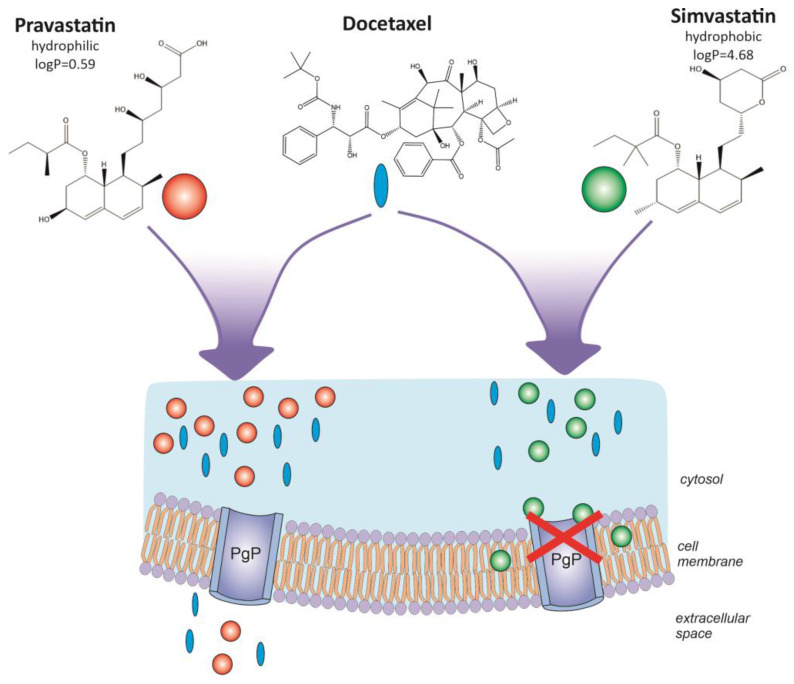
Scheme of action of pravastatin (hydrophilic) and simvastatin (hydrophobic) in the presence of docetaxel.

Atorvastatin increased the docetaxel content of human melanoma cells by an average of two hundred percent over a docetaxel-only control in a radial phase melanoma line (WM35). In melanoma cells from more advanced stages of progression, absorption was 30–45% higher. Similarly, this effect is associated with the strong inhibition of the viability of melanoma cells in all developmental stages by atorvastatin. However, the mechanism of action of atorvastatin may not be related to the ABCB1 channel. There is a chance that its action is related to MRP1 or ABCB5, other ABC cassettes with the ability to eject docetaxel from cells and whose expression has been proven at the mRNA level. Docetaxel retention is a permanent process, ending with the cell entering a state of mitotic catastrophe. A slightly weaker effect of fluvastatin is characterized by correlations between the amount of docetaxel retained in melanoma cells under its influence and cell viability. In both cases, i.e., atorvastatin and fluvastatin, there is synergism in action with the docetaxel; both statins increase the concentration of docetaxel accumulated selectively in human melanoma tumor cells. Rosuvastatin has a weaker effect. Pravastatin, as mentioned, as a highly hydrophilic molecule cannot modify the absorption of docetaxel into tumor cells. Considering the data obtained in the first phase of analysis related to the effect of simvastatin, the data obtained in the second phase of the analysis and taking into account the physicochemical properties of the above-mentioned pharmaceuticals, it should be concluded that statins with similar solubility in aqueous solutions, regardless of their chemical structure, are capable of increasing absorption and the blocking of excretion of docetaxel by human melanoma cells (statin solubility in [mg/mL] in water,: atorvastatin 1.23, rosuvastatin 0.0886, fluvastatin 0.00046, simvastatin 0.0122, and pravastatin as much as 19.0). In this case, the structural similarity of the statins is of secondary importance, which has been demonstrated concerning pravastatin and simvastatin. Both molecules are based on the same structural and spatial motif (Figure 7). 

## 5. Conclusions

The results presented in the manuscript show that hydrophobic statins administered with docetaxel block the ABCB1 channel. In a composition like this, statins increase the accumulation of the drug in the tumor resistance cells and reduce the required doses of cytostatics. Hydrophobic statins in the presence of cytostatics increase their effectiveness while reducing their overall toxicity. This may influence the development of new therapies using hydrophobic statins in oncology therapy.

## 6. Patents

EP, WO, and US patents: EP3570828B1, WO2018135959A1, and US10912756B2.

## Data Availability

No data are available in public data repositories. All data are available in this paper and supplementary materials.

## Supplementary Materials

The following supporting information can be downloaded at: https://www.mdpi.com/article/10.3390/biom13121682/s1. Figure S1: Cell proliferation of a radial melanoma lineage WM35, MTT test; A—Atorvastatin, B—Fluvastatin, C—Pravastatin, D—Rosuvastatin, E—Simvastatin. Statistically significant results are marked with asterisks; Figure S2: Comparison of DNA damage in the comet assay for WM35 melanoma cell line in which different statins were used together with docetaxel. The TM value (*Tail Moment length*) was used for the calculations. An average of 200 cells was used in the experiment. Statnins: A—Atorvastatin, F—Fluvastatin, P—Pravastatin, R—Rosuvastatin, S—Simvastatin and T—Docetaxel; Table S1: The size of human melanoma tumors formed after subcutaneous inoculation of A375P cells in SCID mice in the control, docetaxel, and docetaxel with simvastatin groups. d1, d2, d3—three dimensions of the tumors; Table S2: Comparison of mean size values of melanoma tumors formed after subcutaneous inoculation of A375P cells in SCID mice and individual dimensions (d1, d2, d3) in groups: control, docetaxel, and docetaxel+simvastatin; Table S3 Recovery of docetaxel from three melanoma cell lines (WM 35, WM239A, A375P) from different development stages per million cells and in percentage. Cells were stimulated with docetaxel with the appropriate statin. Docetaxel was isolated from the cells and subjected to HPLC analysis.
