# Supplementary material for: The Blocking of Drug Resistance Channels by Selected Hydrophobic Statins in Chemoresistance Human Melanoma
Source: Biomolecules. 2023 Nov 21;13(12):1682. doi: 10.3390/biom13121682 (PMC10741734; doi:10.3390/biom13121682)
Supplement: Supplementary file 1 [file biomolecules-13-01682-s001.zip › biomolecules-2675835-supplementary.pdf]

SUPPLEMENTARY MATERIALS

Supplementary Methods

Determination of protein concentration

Fluorimetric Qubit® Protein Assay Kit (Invitrogen, Life Technologies, USA) inrelies on binding to proteins of a fluorescent dye specific for them, which when combined shows emission several orders of magnitude higher compared to the free state. Determination of the protein concentration in the tested samples was performed according to the procedure provided by the test manufacturer, which is recommended for this purpose test tubes - Qubit® assay tubes. Fluorescence was measured using a Qubit® fluorometer (Invitrogen, Molecular Probes, USA), previously calibrated according to the manufacturer's instructions, which automatically converted the RFU values to the unit of protein concentration in the sample. Since the presence of compounds used during homogenization, such as 2-mercaptoethanol or SDS, does not affect the determination result, this method was used most often. The tests were performed according to the manufacturer's protocol.

Microscopic test of annexin V-propidium iodide staining of apoptotic cells

Cells were cultured in small plates with a scale for cell counting (Sarsted) for 24 h, then stimulated with drugs at the above-mentioned concentrations for 24 h. After this time, the medium was collected from above the cells very gently, with a capillary tip, until it was completely removed. The test was performed according to the test manufacturer's instructions (Anexin-V-FLUOS Staining Kit, Roche). The excitation wavelength for fluorescein (with annexin) is 488 nm, and the emission maximum is 518 nm. For propidium iodide, respectively: 488-540 nm and 617 nm. Cells not stimulated with the tested drugs were used as a negative control (melanoma cells cultured for 24 hours).

Evaluation of the genotoxicity of the drugs used in melanoma cells

- Comet System 3.0. Quantitative DNA damage analysis

The images of the "comets" were analysed at 200x magnification using an Olympus BX-50 epifluorescent microscope connected to a CCD camera, equipped with a 100W mercury burner, excitation filter 515-560 nm and a barrier filter from 590 nm. In the analysis of nuclear DNA damage, the Komet 3.0 image analysis program by Kinetic Imaging (Liverpool, UK) was used. The basis for the assessment of the most frequently used parameters describing quantitative DNA damage. The following parameters were selected for the assessment of DNA damage: T-DNA (tail DNA) - the percentage of DNA in the tail of the comet. It was determined based on the

fluorescence intensity of DNA located outside the head of the comet. TL (tail length) - determined based on the measurement of the length of the comet's tail, i.e. measurement of the distance of migration of loops and DNA fragments under the influence of the applied electric field from the edge of the head end to the end of the tail. TM (tail moment) - defined as the product of the two above measures.

Analysis of the absorption of pharmaceuticals into human melanoma cells

- Mass spectrometry analysis of selected active compounds.

To verify identity of the compounds used for the experiments, high resolution mass spectrometry was used to confirm MW of the antitumor therapeutics. Exploris 240 HRMS connected via nanoESI ion source to Ultimate 3000 nanoLC (both from Thermo, Bremen, Germany) were used for the confirmation. Samples were introduced in a volume of 1 μl (total 200 fmol/injection) into the PepMap 100 C18 precolumn 500 μm ID/5 mm length. Then samples were washed out from salts and buffer remainings and transferred to Acclaim PepMap C18 column (75 μm ID/150 mm length). Short gradient separation was applied. Solvents used: solvent A 99.9% H_2O + 0.1% HCOOH (v/v), solvent B 99.9% acetonitrile + 0.1% HCOOH (v/v). Flow rate was set to 300 nl/min. Gradient parameters: t=0 min 5%B, t=20 min 40%B, t=21 min 90%B, t=23 min 90%B, t=24 min 5%B, t=26 min 5%B. Mass spectrometer was working in MS only mode with maximal resolution (240 000). Calibration was done the same day using Pierce FlexMix™ Calibration solution (Thermo, Bremen, Germany). Other basic parameters were as follows: ion source used: nanoESI, scanning speed ca. 3 Hz, heated capillary temperature 375°C, scan range 400-1000 m/z. Antitumor agents identity check was done by the comparison between theoretical MW and measured ones. Overlapping of the theoretical and measured peaks were done in Freestyle software (ver 1.8.51.0, Thermo, Bremen, Germany).

***In vivo* studies in SCID mice**

SCID mice implanted with human melanoma tumour cells: A375P, WM239A, and WM35 were used in the planned experiment. Stage I: optimization of the experimental protocol, consisting of the initial analysis of the neoplastic potential of the cells of the tested melanoma lines by determining the kinetics of tumour growth and their ability to metastasize. At this stage, three experimental groups were created and implanted subcutaneously with A375P, WM239A, or WM35 cells (n=6 in each experimental group; total n=18). Based on the results of this stage, one of the three lines was selected and used in further analyses. The selection criterion was (1) the maximum tumour acceptability and (2) the minimum dispersion between the obtained growth kinetics.

Supplementary Figures and Tables

Figures

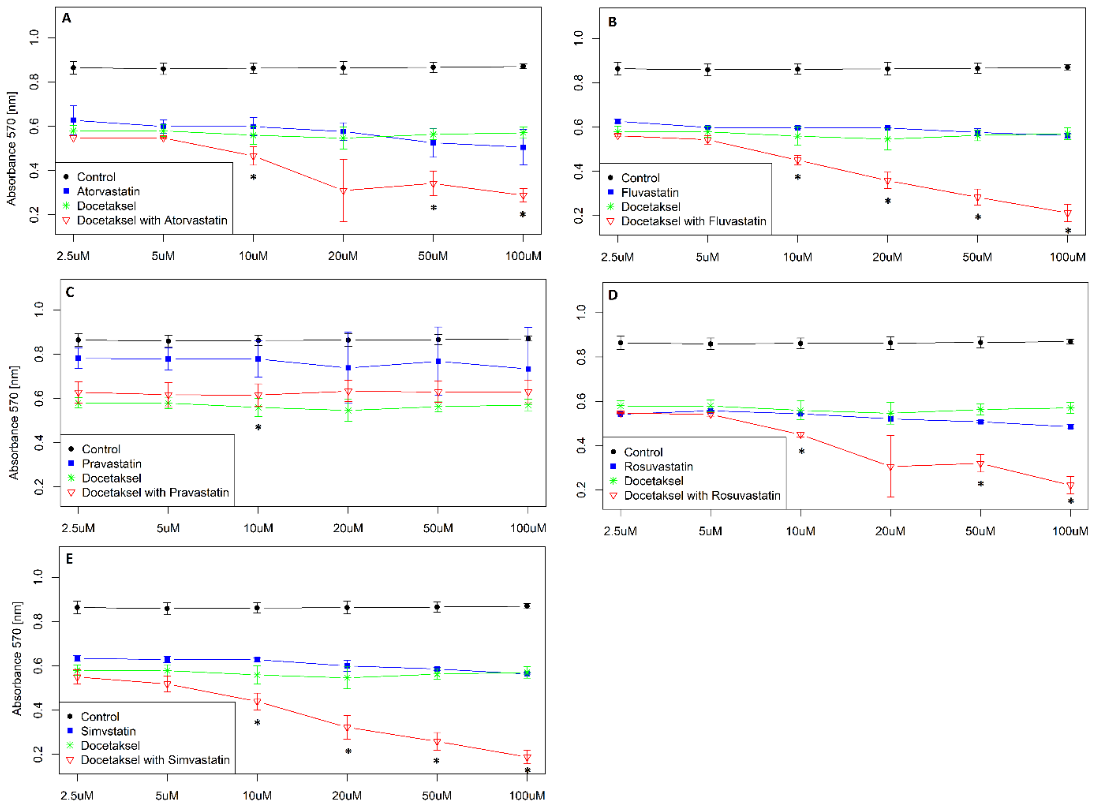

Figure S1. Cell proliferation of a radial melanoma lineage WM35, MTT test; A-Atorvastatin, B - Fluvastatin, C - Pravastatin, D - Rosuvastatin, E – Simvastatin. Statistically significant results are marked with asterisks.

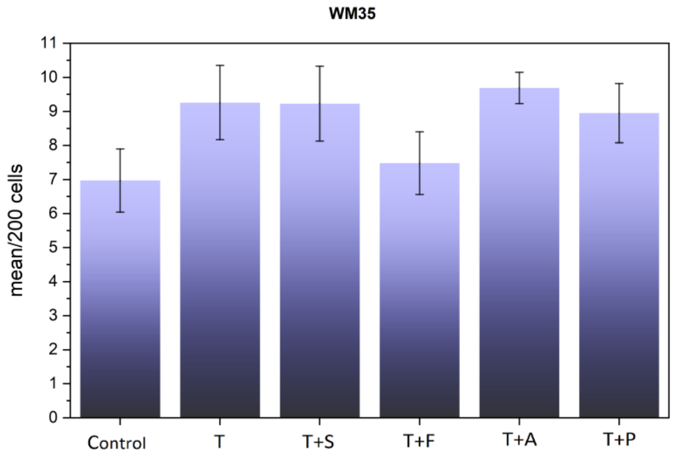

Figure S2 Comparison of DNA damage in the comet assay for WM35 melanoma cell line in which different statins were used together with docetaxel. The TM value (*Tail Moment length*) was used for the calculations. An average of 200 cells was used in the experiment. Statnins: A- Atorvastatin, F - Fluvastatin, P - Pravastatin, R - Rosuvastatin, S – Simvastatin and T – Docetaxel.

Tables

Table S1. The size of human melanoma tumors formed after subcutaneous inoculation of A375P cells in SCID mice in the control, docetaxel, and docetaxel with simvastatin groups. d1, d2, d3 - three dimensions of the tumors.

	variables	n	mean	sd	median	min	max	range
control	d1 [mm]	10	16.28	4.71	16.45	8.74	23.25	14.51
	d2 [mm]	10	12.66	3.18	11.78	8.13	18.91	10.78
	d3 [mm]	10	7.93	1.96	7.71	4.98	10.36	5.38
	size [mm3]	10	1725.43	904.25	1734.52	556.30	3252.20	2695.90
docetaxel	d1 [mm]	15	12.84	3.83	12.30	6,69	18,94	12,25
	d2 [mm]	15	12.09	2.98	12.65	5,71	16.10	10,39
	d3 [mm]	15	10.73	4.28	10.36	4,81	17.00	12,19
	size [mm3]	15	1524.76	1081.89	1261.98	183,74	3924.96	3741,22
docetaxel +simvastatin	d1 [mm]	15	8.26	4.08	7.07	3.08	15.91	12.83
	d2 [mm]	15	6.77	2.82	7.49	3.75	11.50	7.75
	d3 [mm]	15	7.09	4.38	6.20	3.43	18.80	15.37
	size [mm3]	15	639.5	819.73	297.2	40.54	2532.83	2492.29

Table S2. Comparison of mean size values of melanoma tumors formed after subcutaneous inoculation of A375P cells in SCID mice and individual dimensions (d1, d2, d3) in groups: control, docetaxel, and docetaxel+simvastatin.

Variables	*Anova*		*Tukey post hock test*	
	groups	*p-value*	groups compared	*p-value*
Size	• control<br>• docetaxel<br>• docetaxel+simvastatin	0.0106	docetaxel vs control	0.99
			docetaxel+simvastatin vs control	0.03
			docetaxel+simvastatin vs docetaxel	0.02
d1	• control<br>• doceaxel<br>• docetaxel+simvastatin	0.0002	docetaxel vs control	0.09
			docetaxel+simvastatin vs control	0.0001
			docetaxel+simvastatin vs docetaxel	0.02
d2	• control<br>• docetaxel<br>• docetaxel+simvastatin	0.0001	docetaxel vs control	0.60
			docetaxel+simvastatin vs control	0.0003
			docetaxel+simvastatin vs docetaxel	0.001
d3	• control<br>• docetaxel<br>• docetaxel+simvastatin	0.12	docetaxel vs control	0.57
			docetaxel+simvastatin vs control	0.66
			docetaxel+simvastatin vs docetaxel	0.10

Table S3. Recovery of docetaxel from three melanoma cell lines (WM 35, WM239A, A375P) from different development stages per million cells and in percentage. Cells

were stimulated with docetaxel with the appropriate statin. Docetaxel was isolated from the cells and subjected to HPLC analysis.

**Cell line**	**WM 35**		**WM239A**		**A375P**	
**number of cells [mln]**	**35,4**		**34,2**		**22,5**	
	Concentration docetaxel [μM] / million cells (sd)	Content of docetaxel in cells [%]	Concentration taxol [μM] / million cells (sd)	Content of docetaxel in cells [%]	Concentration taxol [μM] / million cells (sd)	Content of docetaxel in cells [%]
**Control**	0	0	0	0	0	0
**(T) Docetaxel**	57.08 (1.61)	100	74.8 (2.18)	100	36 (1.60)	100
**T+Atorvastatin**	112 (3.16)	196	86.7 (2.53)	116	41.4 (1,84)	115
**T+Fluvastatin**	117.9 (3.33)	206	89.3 (2.6)	119	42.4 (1.88)	117
**T+Simvastatin**	131 (3.16)	229	101.87 (2.53)	136	56.50 (1.84)	157
**T+ Rosuvastatin**	120 (3.39)	210	98.8 (2.88)	132	52.2 (2.32)	145
